# Tripartite motif family proteins in inflammatory bowel disease: Mechanisms and potential for interventions

**DOI:** 10.1111/cpr.13222

**Published:** 2022-04-04

**Authors:** Rirong Chen, Yizhe Tie, Jinyu Lu, Li Li, Zhirong Zeng, Minhu Chen, Shenghong Zhang

**Affiliations:** ^1^ Department of Gastroenterology, The First Affiliated Hospital Sun Yat‐Sen University Guangzhou China; ^2^ Department of Clinical Medicine, Zhongshan School of Medicine Sun Yat‐Sen University Guangzhou China; ^3^ Reproductive Medicine Center, Sun Yat‐Sen Memorial Hospital Sun Yat‐Sen University Guangzhou China

## Abstract

Inflammatory bowel disease (IBD) is a chronic recurrent gastrointestinal inflammatory disease that poses a heavy burden to the global healthcare system. However, the current paucity of mechanistic understanding of IBD pathogenesis hampers the development of aetiology‐directed therapies. Novel therapeutic options based on IBD pathogenesis are urgently needed for attaining better long‐term prognosis for IBD patients. The tripartite motif (TRIM) family is a large protein family including more than 70 structurally conservative members, typically characterized by their RBCC structure, which primarily function as E3 ubiquitin ligases in post‐translational modification. They have emerged as regulators of a broad range of cellular mechanisms, including proliferation, differentiation, transcription and immune regulation. TRIM family proteins are involved in multiple diseases, such as viral infection, cancer and autoimmune disorders, including inflammatory bowel disease. This review provides a comprehensive perspective on TRIM proteins' involvement in the pathophysiology and progression of IBD, in particular, on intestinal mucosal barriers, gene susceptibility and opportunistic infections, thus providing novel therapeutic targets for this complicated disease. However, the exact mechanisms of TRIM proteins in IBD pathogenesis and IBD‐related carcinogenesis are still unknown, and more studies are warranted to explore potential therapeutic targets of TRIM proteins in IBD.

## 1 INTRODUCTION

Inflammatory bowel disease (IBD) is a chronic relapsing inflammatory disease of the gastrointestinal tract that can be subdivided into Crohn's disease (CD) and ulcerative colitis (UC).^1^ CD is distinguished by transmural inflammation and skip lesions distributed throughout the gastrointestinal tract, usually complicated by perianal lesions. UC associated inflammation is generally confined to rectum and colon and afflicts only the superficial mucosa, which usually demonstrates mucopurulent bloody stool.^2^, ^3^ The prevalence of IBD is as high as 0.5% in western countries, which has brought huge expenditure and heavy burdens to the healthcare system.^4^ In 2020, the annual direct expenditure of every UC patient in Europe was estimated as €2000, and that of CD was €3500.^5^ Moreover, in newly industrialized countries with lower IBD prevalence, such as China and India, the large populations and rising incidence rates bring remarkably heavy burdens to society.^6^ At present, the mainstream therapy for IBD focuses on immunosuppression, in which biological agents have significantly improved prognosis.^7^ However, despite these advances, over 40% IBD patients still require at least one surgery during the course of the disease; moreover, non‐responses to biologics are reported more often than not.^8^, ^9^ Therefore, new therapies based on a mechanistic understanding of IBD pathogenesis are urgently needed to develop more potent treatment regimens and reap long‐term clinical benefits for IBD patients.

The pathogenesis of IBD remains unclear; it has been generally recognized to be the result of complex interactions between multiple factors, including genetic susceptibility, environmental determinants, intestinal flora and an imbalance of innate and adaptive immunity.^10^ Nowadays, a number of studies have demonstrated that the intestinal barrier performs an essential role in the pathophysiology of IBD.^11^, ^12^ This barrier encompasses four parts, the mechanical, biological, chemical and immune barriers, and IBD are characterized by the impairment of multiple components among them.^13^

The tripartite motif (TRIM) protein family contains more than 70 members characterized by the RBCC structure, in which one or two variable C‐terminal domains show high structural diversity that determines the functional specificity of each protein.^14^ TRIM proteins mainly function as E3 ubiquitin ligases, thus participating in the ubiquitination process and regulating many important post‐translational protein modifications.^15^ TRIM family proteins not only play essential roles in many biological processes, including proliferation, differentiation, transcription and apoptosis, along with the regulation of immune responses, but also participate in many diseases, including cancer, infectious diseases, neurodegeneration and developmental diseases.^16^, ^17^ Recently, TRIM family proteins, such as TRIM20 and TRIM27, were demonstrated to regulate intestinal barrier function and get involved in the pathophysiology of IBD. This review summarizes the structures and functions of TRIM proteins and discusses the mechanisms underlying their role in IBD pathophysiology to provide a novel approach for the exploration of potential therapeutic targets for IBD.

## 2 TRIM FAMILY

### 2.1 Structure of TRIM family proteins

In general, TRIM family members share a highly conservative and typical structure of “RBCC”,^18^ which means that the order of TRIM proteins, from N‐ to C‐terminus, is a really interesting new gene (RING) domain, one or two B‐box domains and a coiled‐coil (CC) domain. The RBCC domain is usually followed by more divergent C‐terminal domains, which determine the specificity of each TRIM protein.^19^ Moreover, the subcellular distributions of TRIM proteins show high variability, with their presence being reported in the cytoplasm, nucleus and plasma membrane.^20^

The RBCC motif, which generally consists of three specific domains, is present in almost all members of the TRIM family.^18^ The RING domain is the foremost structure consisting of 40–60 amino acid residues with the zinc‐finger structure that binds two zinc ions.^17^ Some investigations have found that they specifically bind to E2 ubiquitin‐conjugating enzymes to exert E3 ubiquitin ligase activity, thus mediating the conjugation of specific proteins with one of the most widely recognized post‐translational modifiers, ubiquitin.^17^ The B‐box domain consists of 32–42 residues located behind the RING domain, which can combine with one or two zinc atoms as well.^17^ Depending on the number of residues, it can be divided into B‐Box 1 and B‐Box 2.^21^ Nevertheless, not every TRIM protein contains both simultaneously; some, such as TRIM36, TRIM46 and TRIM54, only have B‐Box 2 domains. B‐Box 1 invariably locates ahead of B‐Box 2 when both exist.^22^ However, the exact functions of B‐box domains remain unclear. Since B‐box shares similar structural characteristics with the RING domain, it is speculated that B‐box may function as E3 ubiquitin ligase, similar to the RING domain. Several previous studies have attempted to support this hypothesis. For example, TRIM16 and TRIM29 still function as E3 ubiquitin ligases even though they lack the RING domain, and further analysis suggested that the B‐box domain shares similar folding pattern and zinc‐binding characteristics with the RING domain, which are important for E3 ligase activity.^23^, ^24^ Moreover, some researchers hypothesize that B‐box enhance the recognition of target proteins.^25^ The coiled‐coil (CC) domain is the third characteristic structure of TRIM proteins, which is known to mediate the homologous or heterogeneous oligomerization of TRIM proteins and has a specific subcellular localization function.^26^

Unlike the highly conserved RBCC sequences, the C‐terminal domain behind the RBCC domain is highly variable; they have been implicated in substrate recognition and serve as a binding site for different targets.^27^, ^28^ So far, a total of ten types of C‐terminal domains have been identified by structural analysis, and different combinations of them allow TRIM proteins to be categorized into 11 distinct subclasses (C‐I to C‐XI in Table 1), which may contain no or as many as three C‐terminal domains.^36^ In addition, a specific set of TRIM proteins which lack the RING domain are classified as the uncategorized group (Table 1).^27^ The most prevalent C‐terminal domain supposes to be SPRY, which exists in more than half of the TRIM family members and sometimes coexists with the PRY domain.^19^ The SPRY domain mediates the recognition of and interaction with target proteins or RNA and regulates host–pathogen interactions and innate immune responses.^37^, ^38^ The C‐terminal subgroup one signature (COS) domain is another frequently encountered C‐terminal domain, which can bind to the microtubule cytoskeleton and participate in homodimerization or heterodimerization.^39^ The fibronectin type 3 (FN3) domain interacts with DNA and heparin, and plant homeodomain (PHD) mediates binding to histones to regulate transcription .^19^, ^32^ The meprin and tumour necrosis factor (TNF) receptor‐associated factor homology (MATH) domain interacts with TNF receptors and regulates the function of important transcription factors, such as NF‐κB.^34^ Less common C‐terminal structures include ADP‐ribosylation factor‐like (ARF), filamin‐type immunoglobulin (FIL), transmembrane (TM), and NHL (named after three proteins, with “H” representing HT2A [TRIM32]) domains. In summary, the high variability of C‐terminal domains determines TRIM's specificity and functional diversity by binding to different substrates, thus exerting diverse regulatory effects.^40^

**TABLE 1:** Classification and structure of TRIM proteins in humans

Family	RBCC structure	Members	Functions
C‐I	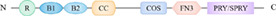	TRIM1, TRIM9, TRIM18, TRIM36, **TRIM46**, TRIM57, TRIM67	Interaction with microtubule cytoskeleton^29^
C‐II	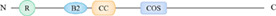	TRIM54, TRIM55, TRIM63	Muscle protein turnover^30^
C‐III	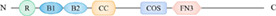	TRIM42	Interaction with DNA and heparin^19^
C‐IV	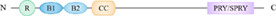	TRIM4, TRIM5, TRIM6, TRIM7, TRIM10, **TRIM11**, TRIM15, TRIM17, **TRIM21**, **TRIM22**, TRIM25, TRIM26, **TRIM27**, **TRIM34**, TRIM35, TRIM38, TRIM39, TRIM41, TRIM43, TRIM47, TRIM48, TRIM49, TRIM50, TRIM51, TRIM53, **TRIM58**, TRIM60, **TRIM62**, TRIM64, TRIM65, TRIM68, TRIM69, TRIM72, TRIM75, TRIM77, TRIML1	Interaction with various proteins or RNA^31^
C‐V	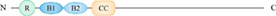	TRIM8, **TRIM19**, **TRIM31**, **TRIM40**, TRIM52, TRIM56, TRIM61, TRIM73, TRIM74	Transcriptional regulation^19^
C‐VI	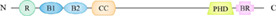	TRIM24, **TRIM28**, **TRIM33**	Interaction with histones and regulation of gene transcription^32^
C‐VII	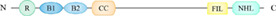	TRIM2, TRIM3, TRIM32, TRIM71	Transcriptional and post‐transcriptional regulation of RNA^33^
C‐VII	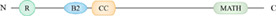	TRIM37	Interaction with TNF receptors and regulation of transcription factors such as NF‐kB.^34^
C‐IX	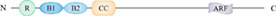	TRIM23	GTPase function^31^
C‐X	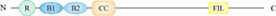	TRIM45	Repressor of multiple signalling pathway^35^
C‐XI	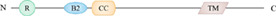	TRIM13, TRIM59	Autophagy modulation^36^
Uncategorized	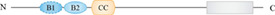	**TRIM14**, TRIM16, **TRIM20**, **TRIM29**, TRIM44, TRIM51, TRIM66, TRIM70, TRIM76	Indirect role in assisting ubiquitination^15^

*Note*: The TRIM proteins associated with IBD or IBD‐related opportunistic infections are shown in bold.

Abbreviations: ARF, ADP‐ribosylation factor‐like domain; B1, B‐Box 1; B2, B‐Box 2; BR, bromodomain; C, C‐terminal; CC, Coiled‐coil Domain; COS, C‐terminal subgroup one signature domain; FIL, filamin‐type immunoglobulin domain; FN3, fibronectin type 3 domain; MATH, meprin, and TNF receptor‐associated factor homology domain; N, N‐terminal; PHD plant homeodomain; R, RING Domain; TM, transmembrane domain.

### 2.2 Functions of TRIM family proteins

TRIM proteins share E3 ubiquitin ligase activity; therefore, they participate in the ubiquitin–proteasome pathway, which is an important post‐translational modification process for regulating the homeostasis and degradation of proteins.^41^

Ubiquitin, a highly conserved protein of 76 residues, is expressed in all eukaryotic cells.^14^ Ubiquitination is an ATP‐dependent process in which the C‐terminal glycine of ubiquitin is covalently bound to a lysine residue of a target protein via the sequential catalysis by an E1 ubiquitin‐activating enzyme, E2 ubiquitin‐conjugating enzyme and E3 ubiquitin ligase.^42^ Afterwards, the ubiquitinated targets are subjected to proteasome degradation, wherein the fate of the substrate is determined by the amount of modified lysine and conjugated ubiquitin.^43^ Depending on the functions of target proteins, ubiquitination participates in many biological processes, such as autophagy, innate immune signalling pathways (e.g., JAK‐STAT3 pathway and TLR‐mediated pathway) and carcinogenesis.^14^ Most TRIMs function as E3 ubiquitin ligases to regulate these biological processes, which are depended on the specific substrates recognized by them. Moreover, some TRIMs regulate these processes by binding to ubiquitin‐like proteins including SUMO^44^ and dNEDD8.^45^ The nine unclassified TRIM proteins lacking the RING domain may indirectly promote ubiquitination or act in conjunction with other TRIM proteins.^15^

As a family characterized by specific structural features rather than functional purposes, TRIM proteins are described as modulators of multiple cellular and physiological activities related to many diseases (e.g., cancer, viral infection and autoimmune disorders) by regulating the activity, stability, degradation, distribution and interaction process of some key proteins.^31^, ^46^

## 3 TRIM FAMILY PROTEINS IN IBD

**FIGURE 1:**
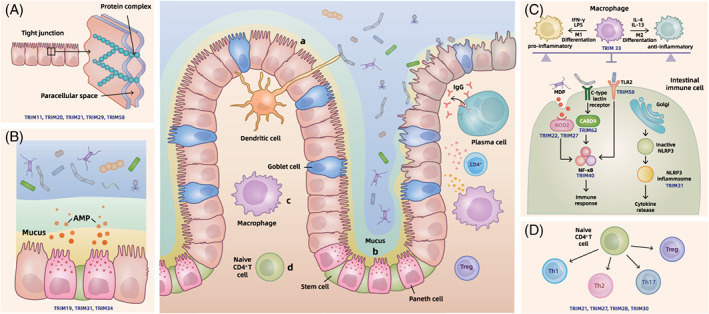
A schematic model of TRIM family proteins in the pathogenesis of IBD. The gut mucosa (in the centre) shows the intestinal condition of healthy state and inflammatory bowel disease (IBD) state. Epithelial cells constitute the major part of this single columnar epithelium, in which goblet cells scatter in them and secrete mucus to form the mucus layer. Paneth cells and stem cells are located at the bottom of the crypts. Dendritic cells can capture and process antigens and present them to T cells. Other immune cells such as macrophage, plasma cells and Treg cells play essential roles in intestinal immune and IBD pathogenesis. IBD‐related intestinal alteration includes the disruption of epithelium and mucus layer, the invasion of luminal microbes and the abnormal activation of immune cells. In each accompanying diagram, IBD‐related TRIMs are listed at the bottom of the schematic model for participation in the shown process. A, Mechanical barrier: Epithelial cell integrity and junctional complexes exert separation roles for the luminal contents, and its impairment may result in the abnormal activation of the intestinal immune. B, Biological and chemical barriers: Trillions of microorganisms colonize in the intestine, and antimicrobial peptides (AMPs) and mucus play protective roles against them, all of which participate in IBD pathogenesis. C, Innate immune: NOD2, CARD9 and TLR2 are essential innate microbial sensors highly correlated with IBD pathogenesis, whose downstream all contains NF‐κB related pathways. Abnormal NLRP3 inflammasome activation and M1/M2 switch in macrophages both participate in the impairment of intestinal immune. D, Adaptive immune: CD^4+^ T lymphocytes differentiate into effector or regulatory T cell subsets to participate in inflammatory and anti‐inflammatory processes

Intestinal barrier dysfunction is an important hallmark of IBD.^47^ TRIM family proteins regulate the function of the four parts of intestinal barrier (mechanical, biological, chemical and immune).These functions are summarized in Table 2 and Figure 1.

**TABLE 2:** Expressions and roles of TRIM family proteins in inflammatory bowel disease

TRIM	Referred barrier functions	Expression in inflammation tissue	Tested cell organizations	Experimental model	Function	Reference
11	Mechanical barrier	—	TRIM11^−/−^ HT‐29 cells and wild type cells; Mice intestinal epithelial cell; active CD patients' colonic mucosa	DSS‐induced colitis mice	TRIM11 participates in TSC1/mTOR's role in restraining epithelial necroptosis and intestinal inflammation by mediating the ubiquitination of RIPK3	48
19	Chemical barrier	—	HeLa cells and HEK293 cells	—	TRIM 19 stimulates human beta‐defensin 2 secretion by regulating myeloid elf‐1‐like factor transcription	49
20	Mechanical barrier	Overexpression	Colon tissues of MEFV^−/−^ and wild type mice	DSS‐induced colitis mice	MEFV regulates epithelial junctional integrity (occluding and claudin‐2), cytokines level (IL‐6, IL‐18) as well as cell markers expression (STAT3)	50
	Immune barrier	Overexpression	Colon tissue of Mefv^V726A/+^, Mefv^V726A/V726A^, TNF^−/−^, TNFR1^−/−^, TNFR2^−/−^ and wild type mice	Mefv^V726A/+^, Mefv^V726A/V726A^ FMF mice model	Mefv^V726A/V726A^ mutation induces substantial colitis with immune cell infiltration in the intestine through regulating the TNF/TNFR axis and TRIM20 (pyrin) activation process	51
	—	Mutation	IBD patient DNA FMF patient DNA	IBD patients	The prevalence of FMF in IBD patients is higher than normal people, and prevalence of IBD in FMF patients is higher as well	52, 53, 54, 55, 56, 57
21	Mechanical barrier; Immune barrier	Downexpression	Intestinal tissue from TRIM21^−/−^ and wild‐type mice Colon tissue from UC‐associated cancer patient	Azoxymethane‐ ‐DSS mice model	TRIM21 can participate in modulating gene expression of Ki67, E‐cadherin, β‐catenin+ cells, matrix metalloproteinase 10, cyclooxygenase 2, hypoxia‐inducible factor‐1α, angiogenin 4, IL‐1β, IL‐6, TNF‐, TGF‐β, Foxp3, IL‐10, IFN‐γ	58
	Immune barrier	Downexpression	TRIM21^−/−^ and wild‐type mice. Inflamed mucosa of patients with IBD	Trinitrobenzene sulfonic induced colitis mice model; CD45RB^high^ CD^4+^ T cell‐induced colitis mice model	TRIM21 restricts intestinal inflammation by inhibiting the differentiation of Th1 and Th17, and its downstream includes interferon regulatory factor 3	59
22	Immune barrier	Mutation	Paediatric‐onset IBD patient gene;	—	Nearly 1% of patients with paediatric‐onset IBD were diagnosed with TRIM22‐related disease	60
	Immune barrier	Mutation	HEK 293 cells; Inflamed and non‐inflamed intestinal and rectal tissues from patients with very early onset IBD;	—	TRIM22 can interact with NOD2 and regulate NOD2‐dependent signalling pathways for various anti‐pathogenic processes (muramyl dipeptide or Respiratory Syncytial Virus)	61
27	Immune barrier	—	Trim27^−/−^ and wild type mice;	DSS‐induced colitis mice	TRIM27 positively regulates activation of STAT3 by promoting JAK1–STAT3 complex formation after IL‐6 stimulation	62
	Immune barrier	Overexpression	HEK293T cells, SW480 cells; CD patients' sigmoid colon tissue	—	TRIM27 restrains NOD2‐mediated inflammatory responses by ubiquitinating and degrading NOD2	63
28	Immune barrier	—	Bone marrow and colon tissues of TRIM28^−/−^ and wild‐ type mice	T cell transfer colitis model	TRIM28 could modulate the epigenetic silencing of Treg‐characteristic genes, thus regulating helper and regulatory T cell differentiation and activation	64
29	Mechanical barrier	—	GHR^−/−^ and wild type mice	Xenograft model	Growth hormone could inhibit DNA repair of epithelial cells by inducing TRIM29	65
30α	Immune barrier	—	LTβR^−/−^, LTαβ^−/−^ and wild type mice	DSS‐induced colitis mice	Lymphotoxin αβ (LTαβ) derived from CD^4+^ T cells binds to lymphotoxin‐β receptor (LTβR) of macrophages to restrict inflammation through TRIM30α‐dependent signal pathway	66
31	Biological barrier	Downexpression	Intestinal tissue of CD patients and controls	—	TRIM31 can enhance the autophagy process in intestinal cells, and this process has no dependencies on Atg5 or Atg7	28
	Immune barrier	—	TRIM31^−/−^ and wild‐type mice	DDS‐induced colitis mice	TRIM31 could ubiquitinate and degenerate NLRP3 inflammasome in macrophages, and TRIM31 deficiency attenuates the colitis severity in symptoms, colonic length, and intestinal histology	67
33	Immune barrier	Downexpression	Trim33^−/−^ and wild‐type mice	DSS‐induced colitis mice	TRIM33 exerts essential roles in intestinal inflammation by modulating recruitment, differentiation, activation, M1/M2 switch processes of macrophages and monocytes in blood and colonic tissues	68
34	Chemical barrier	Downexpression	TRIM34^−/−^, TRIM35^−/−^ and wide type mice; Colonic mucosa from UC patients and healthy subjects	DSS‐induced colitis mice	TRIM34 promotes Mucin 2 exocytosis from colonic goblet cells through the TLR signalling pathway	69
40	Immune barrier	Downexpression	HEK293T, HeLa and SW480 cell lines; Gastrointestinal tissues of humans and mice	—	TRIM40 promotes the neddylation of inhibitor of NF‐κB kinase subunit γ to suppress inflammation in the gastrointestinal tract	45
58	Mechanical barrier; Immune barrier	Downexpression	TRIM58^−/−^ and wide type mice; Surgical resection specimens of active UC patients	DDS‐induced colitis mice	TRIM58 mediates ubiquitination TLR2 and suppresses proinflammatory factors expression to regulate tissue repair and epithelial regeneration, while TRIM 58 deficiency can result in the inflammatory status of the gut	70
62	Immune barrier	—	THP‐1 and HEK293T cells	‐	TRIM 62 could mediate ubiquitination of CARD9, which mediates innate immune and adaptive immune (e.g., NF‐κB signalling and Th1 &Th17 differentiation**)**	71
	Immune barrier	—	TRIM62^−/−^, CARD9^−/−^ and wide type mice	DSS‐induced colitis mice and *C. albicans*‐infected mice	TRIM62 could bind to CARD9 to influence immune response like NF‐κB signalling or MAPK pathway and its deficiency could increase susceptibility to fungal infection	72

### 3.1 TRIM and intestinal barriers

#### 3.1.1 Mechanical barrier

The mechanical barrier, also known as the physical barrier, is an essential defensive line of the intestinal barrier. Its purpose is to separate the luminal contents from the intestinal immune system, whose structural basis is the mucus layer, intact epithelial cells and the junctional complexes between epithelial cells.^73^ Under physiological conditions, the epithelial surface of the intestinal mucosa is covered with mucus, which contributes to both the mechanical and chemical barrier function. The intestinal monolayer epithelium, together with the intercellular junctional complexes (e.g., tight junctions), exerts a significant impact in regulating intestinal permeability and preventing harmful substances (such as bacteria, toxins or other inflammatory mediators) migrating from the intestinal cavity to the epithelium, whose impairments have been reported in the pathogenesis of IBD.^74^, ^75^

TRIM20, encoded by the *MEFV* gene, is widely considered to be associated with the development of familial Mediterranean fever (FMF) .^76^ However, it is frequently reported that FMF patients have a significantly higher incidence of IBD and the incidence of FMF in IBD is also the case in which *MEFV* mutations are often detected (e.g., M694V, M680I and V726A mutation) .^52^, ^53^ Very little is known concerning the underlying mechanism for that phenomenon, although some information has surfaced in the past few years. Sharma et al. found that TRIM20 regulates tight junction (occludin and claudin‐2) integrity, thereby modulating the permeability of the intestinal epithelium in a murine model of dextran sulphate sodium (DSS)‐induced colitis.^50^ Moreover, they found that experimental colitis in homozygous *MEFV* ^*V726A/V726A*^ mutation mice presented much severe colitis, marked by immunocyte infiltration in the intestinal tract, contributing to the immune barrier.^51^ Apart from tight junctions, several TRIM proteins regulate the epithelial integrity to modulate the mechanical barrier function. For example, TRIM11 was reported to be an important modulator of RIPK3‐dependent epithelial necroptosis, whose decline could be observed in the colonic mucosa of CD patients.^48^ In addition, some studies have suggested a protective role of TRIM21 and TRIM58 in IBD pathogenesis via modulating epithelial regeneration, tissue repair and angiogenesis.^58^, ^70^ In addition, Chesnokova et al. observed that growth hormone inhibits epithelial DNA repair by inducing TRIM29.^65^

#### 3.1.2 Biological barrier

The biological barrier is composed of trillions of microorganisms that colonize the intestine. These microorganisms are referred to as the intestinal microflora and could assist in nutrition absorption, host defence and the function of the immune system.^77^ Changes in the composition of intestinal microflora and alterations of interactions between intestinal microflora and intestinal immunity have notable impacts on IBD.^78^ A recent study reported decreased TRIM31 expression in the intestinal tracts of CD patients.^28^ Subsequent experiments revealed that TRIM31 usually accumulates around one type of ubiquitin‐coated invasive bacteria, such as *Shigella* or *Salmonella*. This is attributed to the specific interaction between TRIM31 and the bacterial receptor NDP52, which induces protective autophagy against invasive bacterial infection or other normal commensal bacteria.^28^ Therefore, it was inferred that TRIM31 plays essential roles in restricting invasive bacterial infection in intestinal epithelial cells by promoting autophagy, which can affect gut microbes and lead to IBD in pathological cases.^28^, ^79^

#### 3.1.3 Chemical barrier

The impairment of intestinal chemical barrier, primarily mucus and antimicrobial peptides, can contribute to IBD.^80^ The mucus produced by goblet cells, is rich in mucin glycoprotein, and Mucin‐2 is one of its most abundant components.^12^ Antimicrobial peptides from Paneth cells could promote the permeability of target bacterial membranes and induce bacterial killing.^47^ The chemical barrier is essential for the segregation function and antimicrobial activities in the intestine to implicate the pathogenesis of IBD.^81^

A recent study revealed TRIM34's crucial role in the release of Mucin 2 from goblet cells, which contributes to the integrity of the inner mucus layer and attenuates colitis induced by DSS.^69^ TRIM19 can upregulate the expression of endogenous human β‐defensin 2, which is an important antimicrobial peptide involved in IBD pathogenesis and has been reported to exert protective effects against a variety of pathogens, such as *Escherichia coli* and *Candida albicans*.^49^, ^82^

#### 3.1.4 Immune barrier: adaptive/innate immune

The intestinal microflora, mucosal barrier, and immune system work together to maintain intestinal homeostasis in healthy individuals. However, this balance is disrupted in IBD patients, characterized by dysregulated intestinal flora, destruction of the mucosal barrier, and over‐activation of the immune system.^10^ Studies have shown that both innate and adaptive immunity are engaged in the pathophysiology of IBD.^83^, ^84^ The former is likely to have an effect on IBD through cytokines and complement production, microbial recognition and autophagy, whereas the latter involves the processes of humoral and cellular immunity.^85^ In the following section, we review the association between the immune barrier and TRIM proteins in the pathogenesis of IBD.

#### 3.1.5 Innate immunity

Innate immunity serves as the first line of defence against pathogens and acts by mediating intestinal microorganism recognition and quickly inducing inflammatory responses.^85^ NOD2, CARD9 and TLR2 are some of the crucial molecules involved in innate microbial sensing. The *NOD2* gene, located in the genomic region imparting IBD susceptibility as determined by genome‐wide association studies, encodes NOD2, an intracellular pattern recognition receptor that targets peptidoglycans present in gram‐positive or gram‐negative bacteria, and subsequently activates the NF‐κB signalling pathway.^86^ Both TRIM22 and TRIM27 have been identified as regulators of NOD2 and contribute to intestinal inflammation in IBD.^60^, ^61^, ^63^

CARD9 is another important component of innate microbial sensing pathways identified by exome sequencing for its involvement in IBD, which also lead to the activation of the NF‐κB signalling pathway and cytokine production, especially in fungal infection.^87^ Cao et al. found that TRIM62 only ubiquitinates intact CARD9, while the C‐terminal truncation of CARD9 disrupted this interaction.^72^ As a result, C‐terminal truncated CARD9 variant exhibits protective roles in intestinal inflammation through inhibiting cytokine production and immune response.^72^ This study also demonstrated that TRIM62‐mediated ubiquitinoylation can regulate CARD9‐dependent NF‐κB signalling pathways to alter intestinal immunity in murine models of *Candida albicans* infection and DSS‐induced colitis.^72^ Subsequently, this research team discovered a small molecule targeting for disrupting the interaction between CARD9 and TRIM62, which imitates the protective role of CARD9 variants, to illustrate a novel potential therapy for IBD treatment.^71^ Toll‐like receptor 2 (TLR2) is another pattern recognition receptor, and it has been reported that TRIM58 downregulates innate immune response in myeloid cells via TLR2 signalling pathway, such that severe mucosal injury was observed in TRIM58 knockout mice during DDS induction.^70^ Furthermore, TRIM40 serves as a negative modulator of inflammation that inhibits NF‐κB activity in the gastrointestinal tract.^45^

In addition to microbial recognition, TRIM proteins also participate in IBD pathogenesis by regulating innate immune cells, such as macrophages and myeloid cells.^85^ TRIM33 is essential for monocyte recruitment and differentiation along with macrophage M1/M2 switch and membrane‐bound TNF expression, whose impairment may result in an increase in monocyte and decrease in macrophage counts in the colon accompanied by upregulated colonic inflammation.^68^ In DSS‐induced murine models, TRIM31 was observed to promote the ubiquitin‐proteasome pathway of the NLRP3 inflammasome in macrophages, whose defects have been implicated in IBD pathogenesis.^67^

#### 3.1.6 Adaptive immunity

TRIM family proteins participate in the cellular immunity process of CD^4+^ T lymphocyte differentiation into helper cell types (Th1, Th2 and Th17) and regulatory T (Treg) cells. TRIM21 was observed to have protective roles in IBD by downregulating some pro‐inflammatory cytokines (e.g., IL‐6 and TNF‐α) and inhibiting CD^4+^ T cell differentiation into Th1 and Th17 cells.^58^, ^59^ The IL‐6/JAK/STAT3 signalling pathway has been reported to regulate the differentiation of multiple immune cell types, such as Th2 and Th17 cells.^88^ TRIM27 promotes the interaction between STAT3 and JAK1 in this pathway to trigger STAT3 activation in the serum or colonic mucosa, thereby inducing inflammation.^62^ TRIM27 knockout mice were observed to gain stronger resistance to DSS‐induced colitis, and histopathological examination revealed that their colonic mucosa was more intact, with less disruption.^62^ TRIM28 influences the expansion and differentiation of T cells by silencing Treg‐characteristic genes, thus influencing effector, regulatory and helper T cell phenotypes and autoimmunity in the intestinal tract.^64^ In addition, a TRIM30α‐dependent signalling pathway initiated by the lymphotoxin‐β receptor on macrophages was observed to downregulate the inflammatory response during DSS induction.^66^

## 4 TRIMS AND GENETIC SUSCEPTIBILITY TO IBD

TRIM20, encoded by *MEFV*, is the most‐studied member of the TRIM family that contributes to the genetic susceptibility of IBD.^54^, ^55^, ^56^, ^57^, ^89^, ^90^, ^91^ Recently, it was reported that TRIM20 not only affects the prevalence of IBD but also influences phenotypic characteristics or complications of IBD.^89^, ^90^, ^91^ Several studies have illustrated a correlation between human *MEFV* mutation and paediatric colitis or IBD, among which IBD complicated with FMF is quite common.^54^, ^55^, ^56^, ^57^ Recently, two cases of paediatric IBD were reported in gorillas' model with typical clinical signs, higher levels of C reactive protein and calprotectin, and a clear response to steroids.^92^ Sequencing analysis showed that both of them shared a variant of *MEFV*, which indicates the involvement of TRIM20 in paediatric IBD.^92^ With respect to the association between TRIM proteins and intestinal manifestations, Kasamaki et al. reported one case of *MEFV* mutation with small bowel stenosis, although IBD had not been diagnosed.^93^ Moreover, Fidder et al. found that *MEFV* mutations may be associated with the increased frequency of stricturing patterns and extraintestinal complications in patients with CD.^90^ The E148Q variant of *MEFV* is associated with higher susceptibility to perianal lesions in CD.^91^ Regarding UC, it was reported that *MEFV* mutations may aggravate its clinical progress, and increase the possibility of colectomy.^94^ In addition, Stittrich et al. performed sequence analysis on 38 IBD patients from five families and found that a missense mutation in the PRY/SPRY domain of TRIM11 (p.H414Y) was associated with an increased risk of IBD. The researchers also found that the TRIM11 mutation p.H414Y may increase the activity of the NF‐κB promoter.^95^ NF‐κB is a crucial downstream target in the microbe‐sensing pathways mediated by CARD9 or NOD2, and its activation can promote the expression of multiple proinflammatory cytokines, so as to exacerbate intestinal inflammation and IBD participate in pathophysiology.^87^ Furthermore, TRIM family proteins are critical regulators of the NF‐κB signalling pathway and act by functioning as ubiquitin E3 ligases.^96^ Therefore, the increased risk of IBD in patients with mutant TRIM11 may be related to the activation of the NF‐κB signalling pathway.

## 5 TRIMS AND OPPORTUNISTIC INFECTION IN IBD

A higher risk of severe infections as well as opportunistic infections in IBD patients has been reported, which is generally believed to be a result of the nature of the disease itself or the adverse events associated with the administration of immunosuppressants.^97^ Opportunistic infection can aggravate intestinal inflammation and enhance the prevalence of refractory IBD; thus, some opportunistic pathogens can contribute to IBD pathogenesis or progression.^98^ Human cytomegalovirus (HCMV), *Clostridium difficile (C. diff)*, *Mycobacterium tuberculosis (MTB)* and *Candida albicans* are some of the implicated pathogens, due to their substantial prevalence and incidence.^98^ TRIM family proteins have also been shown to be involved in opportunistic infections (Table 3).

**TABLE 3:** Expression and roles of TRIM family protein in IBD‐related opportunistic infections

TRIM protein	Opportunistic infections	Tested cell organizations	Function	Reference
14	MTB	RAW 264.7 macrophages, HEK293T, murine embryonic fibroblasts, and Lenti‐X cells	TRIM14 negatively regulates macrophages' type I IFN response against MTB infection through a cGAS‐dependent signalling pathway	99
19	HCMV	Human dermal fibroblast and HEK293 and HEK293T cells	HCMV IE1 Protein could bind to and disrupt TRIM19 to gain resistance towards type I interferon‐related immune responses	100
20	C. diff	TNF^−/−^, and TNFR1^−/−^ TNFR2^−/−^ and wild type BM‐derived macrophages	TRIM20 upregulation induced by TcdB can be observed on the basis of TNF/TNFR axis	51
	C. diff	Bone marrow‐derived macrophage and DC 2.4 dendritic cell	TRIM20 binds to inflammasome adaptor ASC to activate caspase 1 inflammasome in response to TcdB	101
	MTB	Samples from FMF patients and healthy controls	FMF patients (over 90% carry MEVF mutations) tend to own stronger host defence against MTB infection	102
22	MTB	HEK 293 cells; Inflamed and non‐inflamed intestinal and rectal tissues from patients with very early onset IBD;	TRIM22 influences the infection of MTB by modulating the NOD2 pathway, NF‐kB pathway, apoptosis, and autophagy in macrophages or monocytes	61
28	HCMV	Human CD34+ cells	The mTOR‐mediated phosphorylation switch of TRIM28 contributes to the eruption of HCMV from the latency period by recruiting HP1α and SETDB1 to the viral genome and inducing the following transcriptional modulation process	103
31	HCMV	Intestinal tissue of CD patients and controls	HCMV‐infected intestinal cells own a lower expression of TRIM31 amount and a higher bacterial load under the suppression of an Atg5/Atg7 independent autophagy pathway	28
46	C. diff	FHC human normal colon epithelial cells; C57/BL6J mice	TcdB induced colonic inflammation through the DUSP1/MAPKs and NF‐κB signalling pathway, in which TRIM46 exerts essential regulatory roles	104
62	Fungus	TRIM62^−/−^, CARD9^−/−^ and wide type mice	TRIM62 ubiquitinates CARD9, modulates the CARD9‐Mediated signalling pathway, and regulates anti‐fungal immunity in the intestine (*C. albicans*)	72

*Note*: C. diff, Clostridium difficile; HCMV, human cytomegalovirus; MTB, mycobacterium tuberculosis; TcdB, Clostridium difficile toxin B.

HCMV usually erupts during the immunosuppressive period of IBD, which is reportedly induced by the mTOR‐mediated TRIM28‐related phosphorylation switch.^103^ Moreover, Ra et al. observed that intestinal cells showed relatively low TRIM31 expression after HCMV infection accompanied by a remarkable bacterial load, which was reversed by TRIM31 reintroduction.^28^ In addition, the HCMV IE1 protein could improve its resistance to antiviral immune responses by binding to and disrupting TRIM19.^100^ Another commonly observed opportunistic infection is caused by *Clostridium difficile* toxin B (TcdB); one of its main virulence factors was reported to induce an inflammatory response in the colon via the TRIM46/DUSP1/MAPKs pathway, and TRIM46 deficiency can restrict colon inflammation.^104^ Conversely, Xu et al. found that TRIM20 mediates the innate immune response to TcdB as a protective role and Sharma et al. further illustrated that TcdB led to the upregulation of TRIM20 expression through the TNF/TNFR pathway.^51^, ^101^ For *MTB*, TRIM22 was shown to modulate the immune response through NOD2 or the NF‐κB pathway, as well as through autophagy.^61^ *MEVF* mutations have been reported to protect against tuberculosis infection,^102^ while TRIM14 functions as an inhibitory modulator of the type I IFN response.^99^ As noted above, TRIM62 regulates anti‐fungal immunity in the intestine by promoting CARD9‐mediated signalling pathway, and its deficiency may increase susceptibility to fungal infections, such as those caused by *Candida albicans*.^72^

## 6 THERAPEUTIC APPLICATIONS OF TRIM FAMILY PROTEINS IN IBD

As noted above, a plethora of TRIM family members have been shown to have protective or detrimental roles in the genetic susceptibility, pathogenesis and complications of IBD.^105^ Efforts have been made to exploit these functions of TRIM proteins in clinical practice.

One group reported that TRIM29 mediates the ubiquitination of stimulator of interferon genes (STING) and the STING‐TBK1‐IRF3 signalling pathway, thereby inhibiting interferon‐I and pro‐inflammatory cytokine production, which contributes to the essential roles of TRIM29 in many autoimmune diseases, including IBD.^105^ Subsequently, this group tried to develop a novel therapy by targeting the inhibition of TRIM29 via gene silencing for diseases such as IBD.^105^

As noted above, TRIM31 downregulates intestinal inflammation in CD by inducing autophagy.^28^ Therefore, compounds comprising TRIM31 and its activator were developed by the same team for treating IBD.^106^ They also proposed the use of TRIM31 for the diagnosis of IBD or screening of IBD therapeutics.^106^ Diagnosis of IBD was achieved by measuring levels of the TRIM31 protein or mRNA in patient intestinal epithelial samples and comparing with control samples, while screening drugs would focus on comparing the expression of TRIM31 when administered or not administered.^106^

## 7 CONCLUSIONS

E3 ubiquitin ligase, which recognizes specific protein substrate and mediates its conjugation with ubiquitin, is a crucial molecule in ubiquitin–proteasome pathway for post‐translational modification, so as to regulate the activity of different proteins in various biological process. The TRIM family is a group of highly conserved proteins with E3 ubiquitin ligase activity, which has been shown to be involved in multiple autoinflammatory diseases, including systemic lupus erythematosus, rheumatoid arthritis and, in particular, inflammatory bowel disease.^107^ A growing number of studies have demonstrated significant differences in TRIM protein expression between IBD patients and the general population, either in the gut or other tissues, suggesting their involvement in IBD. In this review, the relationships between TRIM proteins and IBD were discussed from three aspects: intestinal mucosal barrier function, gene susceptibility and IBD‐related opportunistic infection. TRIM proteins have been shown to modulate the expression of many key molecules in the intestinal mucosal barrier, including tight junction proteins, antimicrobial peptides and mucin. Moreover, TRIM proteins participate in multiple IBD‐related signalling pathways, including microbial recognition (e.g., NOD2, CARD9 and TLR2 pathways), immune cell differentiation (e.g., IL‐6/JAK/STAT3 pathway) and cytokine production (e.g., STING‐TBK1‐IRF3 pathway). In addition, several members of the TRIM family have been implicated in inherited susceptibility to IBD, especially early‐onset IBD, contributing to specific phenotypes and extraintestinal complications. At present, several TRIM proteins (such as TRIM29 and TRIM31) have been proposed for clinical applications, such as diagnosis, treatment and drug screening.

However, there remains much to be learned about TRIM family proteins' effect on IBD and their underlying mechanisms. Although some family members in the C‐I, C‐IV, C‐V, C‐VI, and unclassified subfamilies have been implicated in IBD (Table 1), it is difficult to identify specific regularity between TRIM subfamilies and their roles in IBD, despite the structural and functional similarities within each subfamily. Moreover, most studies evaluating the role of TRIM in IBD have centred on TRIM20—for which retrospective studies usually predominate—while underlying mechanisms remain unclear. Furthermore, the TRIM family is widely accepted as a regulator of tumour oncogenesis or suppression, and its role in colorectal tumours has been reported by several studies.^108^ Nevertheless, few studies on TRIM proteins and IBD‐related carcinogenesis have been reported, so efforts should be made to explore this critical issue. Further clarification of these questions will enable the development of emerging IBD therapies based on the modulation of TRIM family proteins.

## CONFLICT OF INTERESTS

The authors declare that they have no conflicts of interest.

## AUTHOR CONTRIBUTIONS

R.C. and Y.T. wrote and revised the manuscript. J.L. drafted the Figures. L.L., Z.Z., M.C. and S.Z. revised the paper. All authors approved the final vision of the manuscript and the submission.

## Data Availability

DATA AVAILABILITY STATEMENT Data sharing is not applicable to this article, as no new data were created or analyzed in this study.
